# How persons with systemic mastocytosis describe the time between symptom onset and receiving diagnosis

**DOI:** 10.1017/S146342362200024X

**Published:** 2022-09-07

**Authors:** Kerstin Hamberg Levedahl, Annika Nilsson, Birgitta Johansson, Mariann Hedström

**Affiliations:** 1 Department of Public Health and Caring Sciences, Uppsala University, Uppsala, Sweden; 2 Department of Caring Sciences, University of Gävle, Gävle, Sweden; 3 Department of Immunology, Genetics and Pathology, Uppsala University, Uppsala, Sweden

**Keywords:** diagnosis delay, qualitative, rare disease, systemic mastocytosis, systematic text condensation

## Abstract

**Aim:**

The aim of the study was to explore how persons with systemic mastocytosis (SM) described the time between the onset of symptoms and signs and getting the diagnosis.

**Background:**

SM is a rare disease caused by the accumulation of clonal mast cells with abnormal function. The symptoms and signs of the disease are varied, often diffuse and affect individuals differently. Due to this complexity, a multi-disciplinary diagnostic approach is required, in which general practitioners play an important part in identifying and referring patients relevant for such investigations.

**Methods:**

Sixteen persons with SM were interviewed about their experiences of the time before the diagnosis was received. Systematic text condensation was used in the analysis process.

**Findings:**

The time between symptom and signs onset and diagnosis was perceived as difficult. SM often had a complex and unpredictable effect on a person’s daily life, long before diagnosis. In the analysis, three themes were found. *Having symptoms and signs with an unknown cause* included the participants’ descriptions of numerous symptoms and signs, often years before diagnosis. These could be severe and result in worries for both participants and their next-of-kin. *Dealing with the symptoms and signs* encompassed the different ways in which the participants coped with the symptoms and signs, and sought relief. *Being a patient without a diagnosis* underlined the lack of information and knowledge within healthcare, often resulting in a delayed or incorrect diagnosis. The study highlighted the importance of a person-centred approach and the need to increase knowledge of the disease within primary care, to shorten this stressful and vulnerable time.

## Background

Systemic mastocytosis (SM) is a rare disease with an incidence just under one person in 100 000 (van Doormaal *et al.*, 2013; Cohen *et al.,* 2014). SM is caused by the accumulation of clonal mast cells with defective function (Valent *et al.,* 2017). In many cases, SM is difficult to detect due to the range of symptoms and signs caused by the disease. Prolonged timespan between the first symptoms and diagnosis is common for these patients (Gülen *et al.,* 2016; Valent *et al.*, 2017). The diagnosis is determined by a pathological bone marrow sample, mutation D816V in the *KIT* gene and elevated serum total tryptase (Pardanani *et al.*, 2021). In the diagnosis process, a multi-disciplinary investigation is often needed. General practitioners are important to identify and refer patients relevant for such investigations (Valent *et al.*, 2019).

The debut of symptoms and signs often occur between the ages of 20 and 50 years, although the diagnosis is most commonly confirmed between the ages of 40 and 60 years (Gülen *et al.*, 2016). The organs that are most commonly affected by SM are the skin, bones, bone marrow and the gastrointestinal tract. SM is divided into indolent SM (ISM) and advanced SM (AdvSM) (Pardanani *et al.*, 2021). More females are affected by ISM (Cohen *et al.*, 2014; Trizuljak *et al.*, 2020; Fuchs *et al.*, 2021) but slightly more men are affected by AdvSM (Kluin-Nelemans *et al.*, 2021). Survival in ISM is in most cases equal to that in the normal population (Pardanani *et al.*, 2019). The severity of ISM is not dependent on the duration of the disease, nor on the sex or age of the patient (Siebenhaar *et al.*, 2018). AdvSM leads to infiltration of mast cells in organs and causes organ impairment with symptoms such as, e.g., transaminitis, malabsorption and severe osteoporosis (Scherber and Borate, 2018; Wagner and Staubach, 2018). AdvSM is uncommon and usually has a grave prognosis with significantly shortened life expectancy (Pardanani, 2021).

The symptoms and signs can include, e.g., skin and gastrointestinal (GI) problems, cognitive disabilities, depression and life-threatening anaphylactic reactions (Vermeiren *et al.*, 2020; Pardanani *et al.*, 2021). More than 4 of 10 patients have experienced anaphylactic reactions presenting as respiratory problems, low blood pressure, seizures, flush and skin reactions (Gülen *et al.*, 2014). Symptoms and signs can be provoked and aggravated by various individual triggers such as food items, venom (mainly from wasps), stress, alcohol, medication, exercise and high or low temperature (Gülen *et al.*, 2014; Jennings *et al.*, 2018; Valent *et al.*, 2019). Since the disease can give rise to a variety of symptoms and signs and the time between symptom onset and diagnosis can be long, the person’s wellbeing can be severely affected (Jennings *et al.*, 2018). In a case report by Gülen *et al.* (2014), a patient had experienced 97 attacks of varying severity over a period of 25 years before the diagnosis could be set.

Medically unexplained symptoms are defined as physical symptoms extending for weeks without any identified somatic cause (Henningsen *et al.*, 2007). In a recent review, this expression was described as a ‘junk drawer’ that healthcare professionals use when a diagnosis cannot be found for certain symptoms (Rasmussen, 2020). In an interview study, persons with unexplained neurological symptoms described various, often extensive, symptoms that greatly impacted their daily lives. Not having a diagnosis or an explanation for their symptoms further added to their distress, in terms of anxiety and having no sense of the illness trajectory, a feeling of not being ‘legitimately ill’, and anger or despair at being disregarded (Nettleton, 2006). Another study, including patients in primary care with medically unexplained symptoms (Lidén *et al.*, 2015), found similar aspects of distress. In addition, the patients reported how they strived to understand their condition by focussing on their symptoms to describe and manage their situation better. Healthcare professionals were identified as a valuable source of support in this interpretation process (Lidén *et al.*, 2015). The time between symptom onset and getting a diagnosis has been reported as being easier to accept if healthcare professionals listen and are supportive, as well as being honest about lacking knowledge (Huyard, 2009; Litzkendorf et al., 2020).

Studies on the experiences of living with SM are still sparse and, to the best of our knowledge, no studies have focussed specifically on persons with ISM and AdvSM and their experiences before diagnosis. In a recent Danish study based on seven participants with ISM, some participants had experienced being seen as hypochondriacs by healthcare professionals before finally being diagnosed (Jensen *et al.*, 2019). Increased knowledge about how the period from onset of symptoms to diagnosis is experienced may aid the diagnostics of the disease and provide a basis for the development of support interventions. The aim of this study was therefore to explore how persons with SM describe the time between onset of symptoms and signs and receiving the diagnosis.

## Methods

This study had a qualitative explorative design.

### Recruitment and participants

Persons with SM in Sweden can be treated either at a local hospital or be referred to one of two centres for mastocytosis, both located at university hospitals. Participants were recruited from one of these two centres. A purposive sample was used to obtain a variation in age, sex and the two different kinds of SM, ISM and AdvSM. To improve accessibility, only persons living in mid-Sweden were selected for participation in the study, but efforts were made to include persons from both small and large cities. Recruitment was conducted between November 2016 and April 2017. The inclusion criteria were the ability to speak and understand Swedish, being 18 years or older and having had the diagnosis SM confirmed through bone marrow biopsy. The exclusion criteria were severe comorbidity, cognitive impairment/disorder or alcohol/drug abuse. Seventeen eligible persons were approached by healthcare staff at the centre and all expressed interest to participate in the study. These were contacted by phone by the first author and given more information about the study. One participant was excluded due to cognitive impairment evident during the interview. Thus, the study group consisted of 16 participants (Table 1).

**Table 1.** Characteristics for participants (*n* = 16)

Background variables	
**Sex**	
Female	12
Male	4
**Age (years)**	
31–40	1
41–50	1
51–60	1
61–70	8
71–80	3
81–90	2
**Time from first symptoms of disease to diagnosis**	
0–33 years	
**Time from diagnosis to interview**	
0–16 years	
**Type of systemic mastocytosis**	
Indolent	9
Advanced	7

### Interviews

Each participant chose the time and place for their interview. Before the interview started, the participant was asked to read the study information thoroughly and sign the informed consent if they chose to participate. Data were collected using semi-structured interviews. All interviews included questions regarding how it all began, symptoms, signs and triggers and how the various situations were handled. The emotions that these situations aroused in the participants and others around them were also addressed. Probing questions were asked when needed to get a more thorough description. Questions about how the participants described their experience of disease impact on daily life after being diagnosed were also included in the interviews. These results will be presented in a forthcoming publication. The interview guide was initially pilot-tested on one participant who was eligible for the study. No changes were considered necessary and this interview was included in the results. All interviews were performed by the first author and lasted 25–90 min (Md 60 min). Most of the interviews (13) took place at the participant’s home. The other three interviews took place at various locations, chosen by the participants.

### Systematic text condensation analysis

Data were analysed with systematic text condensation, in accordance with Malterud’s description (Malterud, 2012). This is a step-by-step analysis that strengthens the empirical power of a study. The interviews were audio-recorded and thereafter transcribed verbatim. The analysis involved the following steps. The first author read the entire transcripts and four preliminary themes were identified; physical and psychosocial impact of the disease, management and treatment of symptoms, communication with healthcare professionals and deficiencies in care processes. The transcripts were thereafter systematically reviewed to find text connected to the aim of the study, so-called meaning units. Meaning units were marked with a code and sorted into code groups. As the analysis evolved, the names and scope of the code groups were adjusted. Meaning units in each code group of subsections were extracted into a condensate expressing their essence. As the analysis evolved, the names and scope of the code groups were adjusted. In this phase, the preliminary themes were reorganised into three final main themes and seven subsections. Thereafter, quotations that were representative for each subsection’s condensate were selected. The condensates from each subsection were thereafter reconceptualised into an analytic text reflecting the full context. The second and last author listened to random selections of recordings, read parts of the transcripts and participated in the analysis in an iterative process. The amount of empirical data needed to illuminate the aim of the study was considered reached (Malterud, 2012) and the final version of the analysis was approved by all authors. Description of the steps of the analysis process are presented in Table 2.

**Table 2.** The analyse process

Preliminary theme	Example of Meaning unit	Code	Condensate	Subsection	Analytic text	Final theme
Physical and psychosocial impact of the disease	I took a glass of liqueur and felt very sick and woke up outside the toilet	Attacks of mastocytosis	I vomited, got severe diarrhoea, spots, palpitations and became red-skinned in the skin. I could also faint from vigorous exercise, when I ate, drank alcohol but sometimes I didn’t know why. I was scared of the attacks and thought I was going to die of a heart attack or a stroke I couldn’t talk, but just slurred and passed out. The attacks returned several times. Sometimes it took a long time before they passed.	Symptoms, signs and triggers	A serious symptom that was described by both participants with ISM and those with AdvSM was a kind of “attack” that could appear very suddenly, with varying frequency. These attacks could consist of stomach pain, tachycardia, and feeling drained of all power and not being able to communicate with the surroundings, and often resulted in fainting. The duration of the attacks could be hours.	Having symptoms and signs with an unknown cause

## Results

The analysis resulted in three main themes: *Having symptoms and signs with an unknown cause, Dealing with the symptoms and signs* and *Being a patient without a diagnosis.* The themes with associated subsections are presented in Table 3. Quotes to define and enrich the descriptions are included in the results. The symbol /…/ indicates an insignificant excluded statement.

**Table 3.** Main themes and associated subsections

Main themes	Subsections
Having symptoms and signs with an unknown cause	Symptoms, signs and triggers
	Worries: one’s own and others’
Dealing with the symptoms and signs	Self-care
	Seeking information
Being a patient without a diagnosis	Lack of information and coordination
	Being met with belief
	Being erroneously diagnosed

### Theme 1: having symptoms and signs with an unknown cause

#### Symptoms, signs and triggers

The participants described various symptoms and signs that had often appeared years before diagnosis. Many symptoms were related to skin problems, such as rashes, flushes and pruritus. It was common that the rashes grew in number and severity over time. For most participants with ISM, the skin symptoms were the main reason for seeking help from healthcare. Many mentioned that they had some kind of allergy before symptom onset. Some participants had been more tired than usual, and lack of concentration and mood changes were also described.

Most of the participants had experienced GI symptoms before diagnosis. These could consist of vomiting, diarrhoea and stomach pain. Frequent and severe diarrhoea that greatly disturbed everyday life was described as especially problematic by persons with AdvSM, but also by some of the participants with ISM. Almost all participants with AdvSM reported significant weight loss as an early sign of the disease.

I had a lot of diarrhoea before I got the diagnosis, I hardly dared go anywhere, it just ran out of me and I had to use diapers and lost 25 kilos in weight. It took eleven years before they figured out what was wrong (ID 4: AdvSM).

A serious symptom that was described by both participants with ISM and those with AdvSM was a kind of ‘attack’ that could appear very suddenly, with varying frequency. These attacks could consist of stomach pain, tachycardia and feeling drained of all power and not being able to communicate with the surroundings, and often resulted in fainting. The duration of the attacks could be hours.

I’d go out onto the pitch and do a 20-metre rush and suddenly I couldn’t breathe. It was like pushing a button and then like watching a cartoon. All my strength would just disappear, like when you empty a bottle. So I’d get up, I’d take a step but fall to my knees and try to crawl away, then everything turns black and I can’t remember anything more (ID 13: ISM).

The symptoms and signs could be triggered by various kinds of food, exercise, alcohol, sexual activity (including local reactions from semen) and exposure to changes in temperature, e.g., cold/warm weather or a hot shower. However, for many participants, the triggers were not always known. Most participants stated that the symptoms and signs greatly affected their life negatively prior to diagnosis, although some participants had not experienced any major impact on their daily life.

#### Worries: one’s own and others

The occurrence of attacks often resulted in vulnerable and exposed situations. This caused anxiety and fear, in part because the cause of the attacks was unknown. Many feared that they were going to die and struggled to find a reason for the attacks. When the attacks started, some feared that they were suffering from a heart attack or a stroke. The participants also described the worries among family, friends and colleagues, who could become very upset when a participant lost consciousness. Many participants had an ambulance called for them on multiple occasions.

Participants with AdvSM were afraid that their symptoms were caused by some sort of cancer, making the delay in diagnosis hard to bear. The diagnostic procedure could also be very worrying, exemplified in the quotation below.

When the doctor started talking about taking samples for some blood disease, I got really scared, just from hearing the words blood disease, that sounds horrifying, so I got a bit of a panic attack at first and could barely breathe, but then I understood that it wasn’t leukaemia (ID: 12, ISM).

### Theme 2: dealing with the symptoms and signs

#### Self-care

The participants described trying various strategies to manage the symptoms and signs, e.g., dietary changes. Some excluded egg, gluten or milk and some tried something that they described as ‘anticancer food’. Some participants exposed their rashes to the sun to make them paler, although others found that sunlight made the rashes even worse. Non-prescription drugs like antihistamines and different kinds of ointments were also used to manage the symptoms. Thus, the diagnosis could sometimes be delayed by self-treatment.

I talked to a friend and she had stopped eating gluten, so I tried that too and I felt like it helped, that the diarrhoea got a bit better (ID: 7, ISM).

#### Seeking information

As it often took years to get the diagnosis, the participants and their next-of-kin performed searches in literature and on the internet, and asked around to try to find a relevant diagnosis themselves. However, the complex and diffuse symptoms and signs made that difficult. Some concluded that the rashes were due to normal ageing and were harmless. This meant that in several cases participants waited years, even decades, before seeking professional help. Some mentioned feeling embarrassed that they had kept the symptoms to themselves for so long.

My wife, who had been there, sat down and searched to see what it could be and got a hit for anaphylactic reaction. In the morning when I woke up, she said ‘I think I know what you have.’ /…/ and she pulled out a piece of paper and gave it to me. ‘Look at that,’ she said and I recognised all the criteria, I had every single one except that my throat hadn’t swelled up (ID: 13, ISM).

### Theme 3: being a patient without a diagnosis

#### Lack of information and coordination

Almost all participants had sought healthcare for rashes, attacks with affected consciousness or severe diarrhoea and had experienced being told that the cause of the problems was unknown. It was frustrating and exhausting having to fight to get a diagnosis.

I went to the gastro unit and met a skilled physician, but he didn’t understand anything. I don’t know how many gastroscopies I’ve done, they thought it might be reflux or something, but I thought it was obvious that I was allergic to alcohol. But there’s no such disease, so they couldn’t find anything (ID: 15, AdvSM).

The participants also described a lack of coordination in the medical investigations. Referrals could disappear, and there was often a long waiting list for appointments. It often took a long time before the participants were informed about their test results. It was common that many different medical specialities and hospitals were involved in the investigations, which could be confusing. The participants mentioned perceiving that healthcare professionals did not believe that they had the symptoms described, or experiencing that they were stressed, did not provide information and/or had no knowledge about the cause of the symptoms. This created feelings of losing face, being neglected, frustration, sadness, anxiety and anger, and resulted in the conclusion that there was no point in seeking help from healthcare.

Because no-one could give me an answer. I gave them lots of samples of all kinds, but they couldn’t find anything and they didn’t really look either. Since they didn’t know what it was, nothing happened (ID: 5, AdvSM).

Some participants expressed embarrassment at having called the ambulance when no explanation could be found at the hospital. In some cases, the participants put themselves at great risk by choosing to stay at home despite attacks of SM that resulted in a markedly worsening general condition.

I just lay down for a while and then it passed. The doctor had said that I should go to the hospital if I had an attack, but I thought it was so embarrassing to call an ambulance. The attack petered out after a while anyway (ID: 16, ISM).

#### Being met with belief

There were many positive experiences of encounters with healthcare professionals. The participants lauded professionals who took them seriously, listened carefully to their experiences and persisted in searching for a cause for the symptoms and signs. It was generally considered more important to have an empathetic approach and curiosity about what was causing the problems than to have long experience in the profession.

Referrals to specialists were also appreciated.

Yeah, I didn’t know what it was, but later understood that the doctor was very interested in continuing to look for answers, which gave me a sense of security. I mean, I’m a 66-year-old lady with some spots on her head, I didn’t think it was worth bothering about, but she kept looking (ID: 1, AdvSM).

#### Being erroneously diagnosed

The GI problems would sometimes be mistaken for gastrointestinal infection and could lead to an investigation for bowel diseases. The rashes were in several ISM cases assumed to be age-related. That could result in a greatly delayed diagnosis.

I think I got the diagnosis in 2001, but I had been to the healthcare centre in the 90 s and talked about my spots and the doctor had said that they were spots from aging (ID: 18, ISM).

The majority of the participants with AdvSM mentioned first having been told that they had cancer, e.g., colon, bone or breast cancer. This caused them a lot of stress and afterwards led to anger and disbelief.

The doctor said ‘You are so sick, you don’t realise how sick you are.’ We didn’t understand at all, my husband and I. And then he said that we should start with me getting my breasts checked, because it’s in the breasts and I felt like no. And then I got some brochures about cancer and the end of life, and I was supposed to get a cancer nurse as well (ID: 6, AdvSM).

## Discussion

To the best of our knowledge, this is the first qualitative study that focuses on how persons with indolent and advanced SM experience the time before diagnosis. The results provide an understanding of the participants’ life situations, how they managed these situations and their experiences of healthcare prior to getting an explanation for their altered health. Lack of knowledge, delays in getting a correct diagnosis and a mistrusting approach from healthcare were common.

Both ISM and AdvSM participants reported recurrent attacks years before their diagnosis. These attacks could be frightening for both the participants and their next-of-kin. Based on the descriptions, these attacks varied in severity, many resulting in emergency care. Anaphylactic reactions are common among patients diagnosed with SM (Gülen *et al.*, 2014; Abid *et al.,* 2016). However, based on the interview data, it is not possible to determine whether the described attacks met the criteria for anaphylaxis. Such attacks should lead to an investigation for a possible SM diagnosis (Gotlib *et al.*, 2018). Some participants with AdvSM reported having severe symptoms and signs, e.g., severe diarrhoea and loss of weight, years before diagnosis. A delayed diagnosis is especially problematic under such circumstances and reflects that knowledge of the disease is limited in healthcare. Some participants diagnosed with AdvSM had first been given a cancer diagnosis, with a short life expectancy. Among these participants, AdvSM was seen as a non-malignant disease and the diagnosis came as a relief. This is surprising, as AdvSM is also a form of malign diagnosis with a dismal prognosis (Valent *et al.,* 2019; Pardanani, 2021).

Most of the mentioned symptoms, signs and triggers have been reported before (Jennings *et al.*, 2018; Valent *et al.*, 2019¸ Pardanani 2021), although contact with semen during sexual activity has not previously been described as a trigger, to our knowledge. Our results highlighted that the participants themselves managed symptoms and signs of the disease with a number of strategies, such as dietary changes or avoiding exposure to the sun. Participants and their relatives struggled to find answers online and by asking other people if they recognised the, often diffuse, symptoms and signs, which is similar to findings in an earlier study of persons with medically unexplained symptoms (Lidén *et al.*, 2015). Persons with rare diseases often report the internet to be a source of easily accessible information in their search for a diagnosis but also point to the fact that the information found there may not always be reliable (von der Lippe *et al.*, 2017; Littzkendolf, et al. 2020).

After repeatedly seeking care without there being an explanation to the problems, sometimes resulting in feelings of not being believed and of embarrassment, the participants did not see any point in contacting healthcare, instead, they relied on self-care or ignored the symptoms. Several studies report on the importance of getting a diagnosis and an explanation for medically unexplained symptoms (Jennings *et al.*, 2014; Garrino *et al.*, 2015; Walklet *et al.*, 2018; Jensen *et al.*, 2019). Meeting the ‘right’ physician, a physician who was interested in finding a cause for the symptoms the patient presented and who did not stop searching for an explanation, appeared crucial for receiving the correct diagnosis. An interview study of 20 physicians who cared for patients presenting with medically unexplained symptoms revealed a need for education and structure in the care of such patients (Warner *et al.*, 2017). Delay in diagnosis due to lack of knowledge among healthcare professionals is common for many rare diseases (von der Lippe *et al.*, 2017; Llubes-Arrià et al., 2021). For general practitioners, it is a major challenge to have knowledge of rare diseases since, in the Orphanet database, there are 6172 different rare diseases registered (Nguengang Wakapet al., 2020). A recent meta-synthesis (Johansen and Risor, 2017) called for integration of the doctor and patient perspectives and stressed the importance of striving for person-centeredness in physicians’ management strategies.

### Methodological considerations

The first author is a registered nurse with extensive experience working with this patient group and thus has a preunderstanding of SM, but she had not previously met any of the participants. According to Malterud (2001), preconceptions may affect a study in both positive and negative ways. During all steps of the study, the first author made an effort to identify her preconceptions and reflect on these with the other authors. None of the co-authors had any experience of working with this patient group and could, with this naivety, strengthen the reflective process (Malterud, 2001). The study’s trustworthiness (Guba and Lincon, 1991) was strengthened as the transcribed interviews were continuously reviewed, the analysis method was well-established (Malterud, 2012) and the co-authors were all experienced in qualitative analysis.

The participants in this study were positive about sharing their experiences and none declined participation. The notion that the experiences shared were related to SM is supported, as all had their diagnosis confirmed through bone marrow biopsy and none had any severe co-morbidity. The participants included both women and men, representing various ages and places of residence and both ISM and AvdSM. Most of the symptoms and triggers that the participants mentioned have been reported previously. Even though the period during which the participants were diagnosed exceed 15 years, this was a time when the diagnostic and medical procedures for SM were basically unchanged (Arock et al., 2020) and the extended diagnostic period has therefore probably not affected the results markedly. The median length of the interviews was 60 min, which was ample for getting extensive and varied data with high information power. However, some limitations must be addressed. User involvement is important to ensure that patients get the opportunity to influence their care and create confidence in the healthcare. Further user involvement can improve research. In the study planning process, we could have asked persons living with SM to share their perspectives on relevant aspects of the study design, e.g., the interview guide. User reflections and lived experience can also add a valuable layer to the analytic phase (Locock et al., 2019). Involving users in reading and discussing a version of the results before finalisation would probably have strengthened our study’s credibility and transferability.

There was an overrepresentation of female participants. Thus, male perspectives may not be sufficiently covered, thereby possibly lowering both the internal validity and the transferability of the results. It is known that sex inequalities exist in primary care, as women receive fewer examinations and specialist referrals than men (Ballering et al., 2021). Similarly, although the intention was that the sample would consist of variation in the age distribution that reflects the variety in the patient group, there was a greater representation of slightly older persons. Thus, experiences from younger individuals may be underrepresented in the results. However, there is a variety of experiences described and these are valuable regardless of how many participants in different ages that have described them. Those who did not speak Swedish were excluded, which also limited the variation in the sample. Furthermore, the sample consisted mainly of patients with more severe diseases, since all patients had at some point been referred to a university hospital. However, the study’s explorative design means that a description of all possible patient experiences of the subject is not required (Malterud, 2012). The time from symptom onset until diagnosis and the time that the participants had lived with the diagnosis prior to their interview varied. There is a risk that memories of the time between symptom onset and diagnosis in some cases might have changed over time, so-called recall bias (Raphael, 1987). However, it is argued that emotionally significant experiences are remembered more clearly (Roozendaal and McGaugh, 2011). The fact that the participants’ descriptions of the period before diagnosis were detailed and vividly narrated supports the notion that it was well-remembered.

### Clinical implications

General practitioners are often the first to meet patients who present symptoms and signs that are consistent with SM. These symptoms and signs can be diffuse and the patients are at risk for a delayed or missed diagnosis. If not taken seriously, patients might stop seeking medical care. The current study underlines the importance of listening carefully to the patients’ descriptions. When patients describe symptom clusters including some or all of the following; mild to severe gastrointestinal symptoms, pruritus, flushing and skin rashes, allergy with various triggers, recurrent syncope or anaphylaxis, declined cognitive ability or mood swings, general practitioners should consider referring these persons to specialist care for further investigation.

### Conclusion

The time between symptom onset and diagnosis was perceived as difficult, with severe, complex and often unpredictable symptoms that the participants had to deal with on their own, often for many years. The healthcare deficiency in diagnosing and supporting these persons highlights the importance of a person-centred approach and the need to increase knowledge of SM within healthcare, in order to shorten this stressful and vulnerable time.
